# Supplementary material for: The Impact of the Use of Glycomacropeptide on Satiety and Dietary Intake in Phenylketonuria
Source: Nutrients. 2020 Sep 4;12(9):2704. doi: 10.3390/nu12092704 (PMC7576483; doi:10.3390/nu12092704)
Supplement: Supplementary File 1 [file nutrients-12-02704-s001.docx]

**Supplementary table 1a.** Anthropometric measurements weight, height, BMI and Z scores for the CGMP50 group at baseline and 36months.

|  |  | **Baseline** | | | | |  | **36mths** | | | | |
| --- | --- | --- | --- | --- | --- | --- | --- | --- | --- | --- | --- | --- |
| **Subjects male** | **Age** | **Wt kg** | **Wt Z score** | **Ht cm** | **Ht Z**  **score** | **BMI Z**  **score** | **Age** | **Wt kg** | **Wt Z score** | **Ht cm** | **Ht Z**  **score** | **BMI Z**  **score** |
| 1 | 10.1 | 30.93 | 0.56 | 140.00 | 0.16 | 0.68 | 13.7 | 48.73 | 0.85 | 160.40 | 0.36 | 0.93 |
| 2 | 9.4 | 24.82 | -0.26 | 132.50 | -0.55 | 0.04 | 12.8 | 37.04 | -0.11 | 149.00 | -0.56 | 0.27 |
| 3 | 7.5 | 23.25 | 0.64 | 127.00 | 0.43 | 0.54 | 10.5 | 35.81 | 1.02 | 142.10 | 0.23 | 1.28 |
| 4 | 6.9 | 23.71 | 1.29 | 127.30 | 1.14 | 0.94 | 10.1 | 40.68 | 1.74 | 147.00 | 1.26 | 1.71 |
| 5 | 6.4 | 20.28 | 0.68 | 117.70 | -0.16 | 1.09 | 9.7 | 38.24 | 1.67 | 137.50 | 0.14 | 2.16 |
| 6 | 5.8 | 13.40 | -1.93 | 102.70 | -2.52 | -0.06 | 9.1 | 18.08 | -2.37 | 121.20 | -2.19 | -1.20 |
| 7 | 5.1 | 19.69 | 1.49 | 120.30 | 2.18 | 0.20 | 7.9 | 32.91 | 2.09 | 140.70 | 2.47 | 1.39 |
| 8 | 5.3 | 20.95 | 1.83 | 121.00 | 1.96 | 0.98 | 8.3 | 41.14 | 2.73 | 144.00 | 2.51 | 2.43 |
|  |  | **Baseline** | | | | |  | **36mths** | | | | |
| **Subjects female** | **Age** | **Wt kg** | **Wt Z score** | **Ht cm** | **Ht Z**  **score** | **BMI Z**  **score** | **Age** | **Wt kg** | **Wt Z score** | **Ht cm** | **Ht Z**  **score** | **BMI Z**  **score** |
| 1 | 11.6 | 36.82 | 0.27 | 154.60 | 0.97 | 0.39 | 14.7 | 51.30 | 0.32 | 160.40 | -0.19 | 0.60 |
| 2 | 9.2 | 22.33 | -0.91 | 130.70 | -0.57 | 0.88 | 11.9 | 37.81 | 0.03 | 148.90 | -0.10 | 0.06 |
| 3 | 8.4 | 23.59 | -0.01 | 126.20 | -0.60 | 0.40 | 11.4 | 39.00 | 0.60 | 146.40 | 0.00 | 0.79 |
| 4 | 8.1 | 20.09 | -0.86 | 120.80 | -1.29 | -0.18 | 11.1 | 31.47 | -0.26 | 143.70 | -0.14 | -0.34 |
| 5 | 7.6 | 23.93 | 0.60 | 124.40 | -0.07 | 0.86 | 10.7 | 46.41 | 1.72 | 146.40 | 0.59 | 1.94 |
| 6 | 6.2 | 21.22 | 1.04 | 115.80 | -0.17 | 1.47 | 9.4 | 41.38 | 2.00 | 140.20 | 0.88 | 2.17 |
| 7 | 6.2 | 20.05 | 0.68 | 116.40 | 0.09 | 0.82 | 9.2 | 34.59 | 1.28 | 137.90 | 0.63 | 1.35 |
| 8 | 6.2 | 22.03 | 1.45 | 115.90 | -0.10 | 1.95 | 9.4 | 38.74 | 1.73 | 137.60 | 0.44 | 2.03 |

**Supplementary table 1b.** Anthropometric measurements weight, height, BMI and Z scores for the CGMP100 group at baseline and 36months.

|  |  | **Baseline** | | | | |  | **36mths** | | | | |
| --- | --- | --- | --- | --- | --- | --- | --- | --- | --- | --- | --- | --- |
| **Subjects male** | **Age** | **Wt kg** | **Wt Z score** | **Ht cm** | **Ht Z**  **score** | **BMI Z**  **score** | **Age** | **Wt kg** | **Wt Z score** | **Ht cm** | **Ht Z**  **score** | **BMI Z**  **score** |
| 1 | 16.9 | 67.18 | 0.62 | 175.60 | -0.08 | 0.88 | 19.9 | 75.70 | 0.64 | 177.00 | -0.05 | 0.77 |
| 2 | 11.4 | 55.13 | 2.32 | 147.30 | 0.26 | 2.81 | 14.7 | 60.95 | 1.06 | 170.10 | 0.40 | 1.21 |
| 3 | 13.8 | 39.26 | -0.52 | 158.10 | -0.37 | -0.54 | 16.8 | 55.89 | -0.31 | 162.40 | -1.82 | 0.90 |
| 4 | 10.0 | 26.45 | -0.25 | 138.50 | 0.00 | -0.45 | 13.0 | 45.77 | 0.72 | 160.20 | 0.67 | 0.50 |
| 5 | 8.8 | 24.22 | 0.04 | 131.70 | 0.00 | 0.02 | 11.7 | 39.98 | 0.88 | 153.10 | 0.09 | 0.62 |
| 6 | 8.7 | 28.94 | 0.97 | 136.90 | 0.90 | 0.74 | 11.7 | 33.25 | -0.11 | 152.00 | 0.72 | -0.88 |
| 7 | 7.7 | 21.23 | -0.06 | 123.90 | -0.44 | 0.26 | 10.7 | 36.38 | 1.15 | 145.60 | 0.55 | 1.26 |
| 8 | 6.1 | 17.66 | -0.04 | 115.50 | -0.17 | 0.07 | 9.0 | 32.80 | 1.42 | 137.10 | 0.68 | 1.54 |
|  |  | **Baseline** | | | | |  | **36mths** | | | | |
| **Subjects female** | **Age** | **Wt kg** | **Wt Z score** | **Ht cm** | **Ht Z**  **score** | **BMI Z**  **score** | **Age** | **Wt kg** | **Wt Z score** | **Ht cm** | **Ht Z**  **score** | **BMI Z**  **score** |
| 1 | 9.4 | 29.49 | 0.43 | 136.50 | 0.28 | 0.40 | 12.2 | 50.76 | 1.39 | 157.70 | 0.90 | 1.25 |
| 2 | 9.3 | 31.36 | 0.81 | 134.00 | -0.09 | 1.16 | 12.3 | 49.41 | 1.24 | 156.50 | 0.69 | 1.18 |
| 3 | 9.2 | 25.95 | -0.10 | 127.80 | -1.02 | 0.58 | 12.2 | 37.60 | 0.08 | 145.60 | -0.73 | 0.58 |
| 4 | 8.2 | 24.54 | 0.36 | 123.90 | -1.04 | 1.16 | 11.3 | 44.91 | 1.39 | 146.60 | 0.05 | 1.78 |
| 5 | 5.4 | 17.15 | 0.68 | 114.00 | 0.72 | 0.33 | 8.4 | 26.10 | 0.48 | 133.90 | 0.72 | 0.16 |

**Supplementary table 1c.** Anthropometric measurements weight, height, BMI and Z scores for the AA group at baseline and 36months.

|  |  | **Baseline** | | | | |  | **36mths** | | | | |
| --- | --- | --- | --- | --- | --- | --- | --- | --- | --- | --- | --- | --- |
| **Subject male** | **Age** | **Wt kg** | **Wt Z score** | **Ht cm** | **Ht Z**  **score** | **BMI Z**  **score** | **Age** | **Wt kg** | **Wt Z score** | **Ht cm** | **Ht Z**  **score** | **BMI Z**  **score** |
| 1 | 14.2 | 38.06 | -1.07 | 168.20 | 0.49 | -2.52 | 17.7 | 53.60 | -1.27 | 179.30 | 0.34 | -1.82 |
| 2 | 14.4 | 66.58 | 1.63 | 167.00 | 0.20 | 1.98 | 17.0 | 89.00 | 2.60 | 176.40 | -0.04 | 2.70 |
| 3 | 14.1 | 54.68 | 0.86 | 170.80 | 0.94 | 0.54 | 18.0 | 84.70 | 1.97 | 175.10 | -0.30 | 2.25 |
| 4 | 14.0 | 52.57 | 0.78 | 159.50 | -0.32 | 1.32 | 17.9 | 62.76 | 0.17 | 159.50 | -1.15 | 1.04 |
| 5 | 12.6 | 44.76 | 0.91 | 150.30 | -0.26 | 1.41 | 15.5 | 69.57 | 1.34 | 172.70 | 0.15 | 1.66 |
| 6 | 10.4 | 30.20 | 0.16 | 142.10 | 0.24 | 0.02 | 13.6 | 51.72 | 0.93 | 164.50 | 0.60 | 0.88 |
| 7 | 9.8 | 22.70 | -1.08 | 130.30 | -1.16 | -0.56 | 12.9 | 38.84 | 0.00 | 155.40 | 0.19 | -0.21 |
| 8 | 9.1 | 28.72 | 0.84 | 133.40 | -0.04 | 1.20 | 12.1 | 42.78 | 1.00 | 149.90 | 0.12 | 1.30 |
| 9 | 9.0 | 35.19 | 1.68 | 133.50 | -0.31 | 2.24 | 11.9 | 48.89 | 1.40 | 148.40 | 0.07 | 1.85 |
| 10 | 6.4 | 17.86 | -0.29 | 122.00 | 0.65 | -1.19 | 9.5 | 25.94 | -0.08 | 139.40 | 0.60 | -0.68 |
| 11 | 5.9 | 23.48 | 1.97 | 117.10 | 0.33 | 2.42 | 9.2 | 43.35 | 1.76 | 141.50 | 0.02 | 2.32 |
|  |  | **Baseline** | | | | |  | **36mths** | | | | |
| **Subject**  **female** | **Age** | **Wt kg** | **Wt Z score** | **Ht cm** | **Ht Z**  **score** | **BMI Z**  **score** | **Age** | **Wt kg** | **Wt Z score** | **Ht cm** | **Ht Z**  **score** | **BMI Z**  **score** |
| 1 | 15.7 | 74.57 | 1.65 | 163.40 | 0.97 | 1.40 | 18.8 | 72.36 | 1.95 | 165.10 | 0.24 | 1.96 |
| 2 | 13.9 | 80.23 | 3.05 | 175.90 | 2.52 | 2.18 | 17.0 | 69.96 | 1.75 | 177.60 | 2.33 | 0.89 |
| 3 | 13.1 | 50.61 | 1.01 | 163.90 | 1.14 | 0.63 | 16.0 | 54.75 | 0.51 | 166.50 | 0.54 | 0.95 |
| 4 | 11.5 | 40.21 | 0.75 | 160.20 | 1.84 | -0.23 | 14.5 | 51.14 | 0.56 | 167.30 | 0.94 | 0.24 |
| 5 | 11.1 | 41.91 | 1.12 | 148.00 | 0.47 | 1.22 | 14.3 | 54.00 | 0.82 | 161.70 | 0.18 | 0.95 |
| 6 | 8.2 | 19.48 | -0.89 | 126.90 | 0.00 | -1.05 | 11.1 | 31.56 | -0.18 | 148.60 | 0.54 | -0.78 |
| 7 | 5.9 | 24.76 | 1.29 | 114.20 | -0.26 | 1.90 | 8.9 | 36.17 | 2.41 | 131.60 | 0.37 | 2.85 |
| 8 | 5.1 | 18.64 | 1.66 | 108.30 | -0.14 | 2.22 | 7.9 | 37.42 | 1.68 | 128.90 | -0.15 | 2.24 |

Additional technical information on analysis of blood phenylalanine measurement.

The amino acids: phenylalanine tyrosine are measured in underivatised solvent extracts from dried blood spots using a Waters Xevo TQD tandem mass spectrometer with direct injection liquid chromatography sample induction. Multiple reaction monitoring (MRM) acquisition mode is used and analysis is restricted to the specified analytes. Amino acids lose formate following collision-induced dissociation with argon in the collision cell.  Formate has a mass of 46 Da and therefore the amino acids can be detected using a neutral loss of 46. Amino acids are eluted into microtitre plates from dried blood spots with a methanolic solution containing the deuterated internal standards phenylalanine-d5 and tyrosine-d4. The MassLynx software reports the ratio of the analyte peak height to that of its deuterated internal standard. From the ratio the concentration of the amino acid in each sample can be determined using a calibration factor based on the internal standard solution.

The methodology is UKAS accredited to ISO 15189:2012 and included in the Labs scope (Lab 9948)
